# Correcting Task fMRI Signals for Variability in Baseline CBF Improves BOLD-Behavior Relationships: A Feasibility Study in an Aging Model

**DOI:** 10.3389/fnins.2020.00336

**Published:** 2020-04-30

**Authors:** Venkatagiri Krishnamurthy, Lisa C. Krishnamurthy, Jonathan H. Drucker, Suprateek Kundu, Bing Ji, Kyle Hortman, Simone R. Roberts, Kevin Mammino, Stella M. Tran, Kaundinya Gopinath, Keith M. McGregor, Amy D. Rodriguez, Deqiang Qiu, Bruce Crosson, Joe R. Nocera

**Affiliations:** ^1^Department of Neurology, Emory University, Atlanta, GA, United States; ^2^Center for Visual and Neurocognitive Rehabilitation, Atlanta Veterans Affairs Medical Center (VAMC), Decatur, GA, United States; ^3^Department of Physics and Astronomy, Georgia State University, Atlanta, GA, United States; ^4^Department of Biostatistics and Bioinformatics, Emory University, Atlanta, GA, United States; ^5^Department of Radiology and Imaging Sciences, Emory University, Atlanta, GA, United States; ^6^Department of Psychology, Georgia State University, Atlanta, GA, United States; ^7^Division of Physical Therapy, School of Medicine, Emory University, Atlanta, GA, United States

**Keywords:** language fMRI, domain-general, semantic fluency, BOLD deactivation, cerebral blood flow, sensitization, aging

## Abstract

Blood Oxygen Level Dependent (BOLD) functional MRI is a complex neurovascular signal whose magnitude depends on baseline physiological factors such as cerebral blood flow (CBF). Because baseline CBF varies across the brain and is altered with aging, the interpretation of stand-alone aging-related BOLD changes can be misleading. The primary objective of this study was to develop a methodology that combines task fMRI and arterial spin labeling (ASL) techniques to sensitize task-induced BOLD activity by covarying out the baseline physiology (i.e., CBF) in an aging model. We recruited 11 younger and 13 older healthy participants who underwent ASL and an overt language fMRI task (semantic category member generation). We measured in-scanner language performance to investigate the effect of BOLD sensitization on BOLD-behavior relationships. The results demonstrate that our correction approach is effective at enhancing the specificity and sensitivity of the BOLD signal in both groups. In addition, the correction strengthens the statistical association between task BOLD activity and behavioral performance. Although CBF has inherent age dependence, our results show that retaining the age factor within CBF aides in greater sensitization of task fMRI signals. From a cognitive standpoint, compared to young adults, the older participants showed a delayed domain-general language-related task activity possibly due to compromised vessel compliance. Further, assessment of functional evolution of corrected BOLD activity revealed biphasic BOLD dynamics in both groups where BOLD deactivation may reflect greater semantic demand or increased premium on domain general executive functioning in response to task difficulty. Although it was promising to note that the predictability of behavior using the proposed methodology outperforms other methodologies (i.e., no correction and normalization by division), and provides moderate stability and adequate power, further work with a larger cohort and other task designs is necessary to improve the stability of predicting associated behavior. In summary, we recommend correction of task fMRI signals by covarying out baseline CBF especially when comparing groups with different neurovascular properties. Given that ASL and BOLD fMRI are well established and widely employed techniques, our proposed multi-modal methodology can be readily implemented into data processing pipelines to obtain more accurate BOLD activation maps.

## Introduction

Blood Oxygen Level Dependent (BOLD) functional Magnetic Resonance Imaging (fMRI) is a widely used non-invasive neuroimaging technique to study human brain function but can be prone to misinterpretations due to its neurovascular origin (Liu et al., 2013). Although BOLD fMRI is widely regarded as a measure of neural activity, it reflects changes in other physiological variables such as cerebral blood volume (CBV), cerebral blood flow (CBF), and cerebral metabolic rate of oxygen (CMRO_2_) (van Zijl et al., 1998; Hua et al., 2011; Kim and Ogawa, 2012; Blockley et al., 2013), but most notably CBF (Buxton, 2012). Further confounding factors such as medication, age, and disease are also likely to introduce significant variability in the resting physiology resulting in variability of the BOLD response (Liau and Liu, 2009). In the context of aging and aging-related cognitive changes, the influence of baseline CBF on task fMRI BOLD measures and its influence on BOLD-behavior relationships is not well understood. Thus, to better interpret task fMRI signals across different age groups, it is important to remove confounds such as inter-subject variability in baseline CBF, thereby improving sensitivity to task-induced neural activity.

Brain’s basal metabolism is known to account for 20% of the body’s oxygen consumption (Shulman et al., 2004) and thus requires an adequate blood supply to support neural activity (Raichle et al., 2001). The association between BOLD and CBF not only varies across the brain due to its dependency on vessel size (wherein BOLD fluctuations at sites of medium to small vessels were more closely regulated by the dynamic regulation in baseline CBF (Tak et al., 2014), but can also vary across the subjects. Given that various tasks involve cortical and deeper sub-cortical areas that encompasses various vessel size, and task-induced BOLD signal incorporates the influence of baseline CBF (Davis et al., 1998), a more complete understanding of how the variability in baseline CBF across subjects influences the change in task-induced BOLD activity is needed.

A common finding from aging-related task fMRI literature is that older adults show increased frontal BOLD activity (Daselaar et al., 2003; Park et al., 2003; Cabeza et al., 2004; Gutchess et al., 2005), and decreased posterior BOLD activity (Buckner et al., 2000; Daselaar et al., 2003; Park et al., 2003; Cabeza et al., 2004; Gutchess et al., 2005; Davis et al., 2008). It is also well-accepted that aging is associated with vascular changes (O’Rourke and Hashimoto, 2007; Samanez-Larkin and D’Esposito, 2008; Chen et al., 2011; Lu et al., 2011; Gauthier and Hoge, 2013; De Vis et al., 2015), marked by aging-related decline in CBF (Lu et al., 2011). Since BOLD and CBF parameters change spatially with age, the interaction between task-induced BOLD fMRI and baseline physiology (such as CBF) is not well defined.

In the realm of language function, age-related decline in word retrieval is also a frequently observed phenomenon (Burke and Shafto, 2004). The few task fMRI studies that have addressed language production mechanisms (Meinzer et al., 2009; Shafto et al., 2010; Soros et al., 2011) have shown increased frontal activation in older adults (Wierenga et al., 2008). In the context of overt word production tasks, aging-related differences in biphasic BOLD hemodynamic response function (HRF) have been reported by a previous study (Wierenga et al., 2008). However, it is unclear whether the previously reported age-related increase in frontal activity, and language-specific biphasic BOLD activity were more weighted by task-specific neural changes or neural changes masked by baseline physiology. Further, potential decay in task performance can be expected within each block especially if the task is manipulated for difficulty (Meinzer et al., 2012a). Thus, parsing out sensitized BOLD temporal dynamics to understand the functional evolution (Patel et al., 2015) is necessary to tease apart the age-related differences in task-induced neural activity.

The primary objective of this study was to develop a multi-modal MR approach that combined task fMRI and arterial spin labeling (ASL) techniques to sensitize the task-induced BOLD activity to underlying neural substrates by removing the baseline physiological (i.e., CBF) variability in an aging model. Specifically, we focused on exploring whether the proposed group-level normalization by co-variate approach sensitizes the task-BOLD signals better than the widely used normalization by division approach (Bandettini and Wong, 1997). The second objective was to investigate task-specific BOLD dynamics as a function of task evolution (i.e., serial and cumulative presentation of stimuli as a function of time). The third objective was to report the language-specific aging-related neurophysiological and cognitive findings as observed from the sensitized task fMRI activity. We hypothesize that a multimodal (combined task fMRI and ASL) approach is able to increase the sensitivity of task-induced BOLD signal to behavior by removing the influence of baseline CBF on BOLD signal. If our hypotheses are indeed supported this would have implications in the interpretation of fMRI and could lead to additional research aimed to better understand brain physiology and neurovascular dynamics with a focus on cognition and behavior.

## Materials and Methods

### Participants

Study participants were recruited from a volunteer database or community flyer. To meet inclusion criteria, participants had to be between 18 and 34 or 60 and 89 years of age, have no history of depression or neurological disease, report being right handed, and be a native English speaker. Exclusion criteria included conditions that would contraindicate an MRI scan, hospitalization within the past 6 months, or significant cognitive impairment defined as a score < 24 on the Montreal Cognitive Assessment (MoCA). All participants provided informed consent in a process that was approved by the Emory University Institutional Review Board and Atlanta VA Research Oversight committee. All consent procedures were in compliance with the Declaration of Helsinki. We enrolled 43 participants, who completed two study sessions: a cognitive testing session and an MRI session. Nineteen participants were dropped from this study (13 incomplete fMRI and/or ASL datasets due to technical (sequence related) difficulties, and 6 completed datasets that did not meet the quality assurance standards for fMRI and ASL). Therefore, our results are based on 24 participants, *N* = 11 younger adults, *N* = 13 older adults. Table 1 summarizes the demographic characteristics of our sample demonstrating that our groups are well balanced for gender, education, and global cognitive functioning.

**TABLE 1:** Participant demographics and psychological data.

	**Young**	**Old**
	**(*N* = 11)**	**(*N* = 13)**
Age	23.52 ± 3.08	66.86 ± 4.37
Female, *N*	5	5
Years of education	15.82 ± 1.54	15.92 ± 1.63
MoCA	28.27 ± 1.74	27.85 ± 1.63

**MoCA, Montreal Cognitive Assessment; the data is presented as mean ± std.**

### Magnetic Resonance Imaging (MRI) Acquisition

MRI scans were acquired on a 3 Tesla Siemens Prisma MRI scanner (Erlangen, Germany) using the body coil for radio frequency (RF) transmission and a 64-channel phased-array head coil for RF receiving. The participant’s head was comfortably stabilized using foam pads to minimize motion during and between scans. A high-resolution T1-weighted anatomical image for spatial normalization to MNI template space was acquired with a T1w-MPRAGE sequence (TR = 2530 ms, TE = 2.96 ms, TI = 1100 ms, FA = 7°, isotropic resolution = 1 × 1 × 1 mm^3^). To identify areas of activation during overt word generation, three runs of a sparse-sampled (Perrachione and Ghosh, 2013)task fMRI time course was acquired with a BOLD weighted single shot gradient recalled echo planar imaging (EPI) multi-band sequence (FoV = 220 × 220 mm^2^, multi-band acceleration factor = 6, matrix = 100 × 100, 72 slices, interleaved axial acquisition, slice thickness = 2 mm, repetition time (TR) = 4000 ms (1000 ms image acquisition + 3000 ms delay during which participants were cued to make an overt response), echo time (TE) = 33 ms, acquisition bandwidth = 2500 Hz/px, flip angle (FA) = 90°, and 78 measurements per run). To correct for EPI geometric distortions, a pair of spin echo EPI scans with opposite phase encoding directions (“topup”) (Andersson et al., 2003) were acquired that were designed with the same echo spacing and bandwidth as the task fMRI [echo spacing (ES) = 0.52 ms and bandwidth (BW) = 2500 Hz/px]. A single-band reference BOLD EPI scan was acquired immediately prior to the task fMRI time course to facilitate EPI co-registration to T1w-MPRAGE.

A 2D pseudo-Continuous Arterial Spin Labeling (pCASL) EPI sequence to measure resting CBF was acquired with the following parameters: FOV = 220 × 220 mm^2^, matrix = 64 × 64, TR = 4080 ms, TE = 13 ms, GRAPPA factor = 2, twenty 5 mm axial slices in ascending order with a 1 mm gap, post-labeling delay (PLD) = 1.8 s, labeling time = 1500 ms, 47 pairs of label and control acquisitions with a total scan time of 6 min 36 s. A fully relaxed proton density weighted scan (M0) with similar parameters except for TR = 10 s with 2 averages were acquired to convert the perfusion signal to absolute CBF value.

### Task Design

To assess the participant’s brain activity during word retrieval, fMRI data was collected during an overt category member generation task. During the task, a category (e.g., “birds”) was visually displayed at the center of an MR compatible video monitor in lowercase Arial font, and upon reading the category, the participants were asked to generate a word describing an exemplar (e.g., “hawk”) associated with that category, within 3 s of the stimulus presentation. During the 1sec EPI acquisition, the written category was switched to a fixation cross to warn the participants to remain still and silent. Each task run consisted of six categories that were presented in blocks of 8 followed by jittered blocks of 3–5 TRs for control condition (reading the word “rest” aloud) that afforded contrast between semantic engagement against motor speech production. All responses were transcribed on-line by hand and recorded using a MR compatible microphone (OptoAcoustics Inc., Israel) affixed to the head coil. Before scanning, the participants were trained on a practice task set. Participants were instructed to generate a single exemplar every time a category was displayed, remain silent while the fixation cross appeared on the screen, refrain from repeating exemplars, and generate the word “pass” if unable to generate an exemplar within 3 s. The MRI session included 3 runs of 6 blocks requiring generation of six category members, for a total 144 trials. Categories remained constant within blocks but changed between blocks.

### In-Scanner Behavior Analysis

For a given category, the participants’ responses were compared against a library of responses and automatically scored using Microsoft Office EXCEL (Microsoft, WA, United States). An incorrect response was defined as any response not matching the library of correct responses, for example, a semantically unrelated utterance (e.g., − “celery” for BEVERAGES), a filler word (e.g., − “um, er”), no response, a repeated response, or a response of “pass.” The library of category members that defined correct responses consisted of semantically related members of the provided categories (e.g., − “iced tea” for BEVERAGES category). To assess the evolution of responses across each block, accuracy was computed for the first 4 words and the last 4 words separately. That is, each segment (Seg1 = first 4 words, Seg 2 = last 4 words) had a total of 72 trials (4 words ^∗^ 6 categories ^∗^ 3 runs). Percent (%) accuracy for each segment was calculated as a ratio of number of correct trials to total number of trials (72 trials).

### Image Processing

#### Task-fMRI Pre-processing

The BOLD EPI images were processed systematically with a combination of AFNI (Cox, 1996), FSL (Smith et al., 2004), ITK Snap^1^, and Matlab (Natick, MA, United States) in-house scripts. Quality control (QC) of the unprocessed task fMRI time series was carried out using FBIRN (Keator et al., 2016). Only the data sets (i.e., 24 out of 43 participants) that passed QC criteria for both task-fMRI (described in Supplementary Section S1) and CBF were promoted for further processing. All included task fMRI datasets were corrected for slice timing, bulk head motion, and EPI distortion (Andersson et al., 2003). In parallel, the T1w-MPRAGE images were skull stripped using optiBET (Lutkenhoff et al., 2014), and spatially transformed to MNI-152 standard template using FSL’s linear (FLIRT) and non-linear (FNIRT) spatial transformation algorithms. The EPI-distortion corrected BOLD images were co-registered with the T1w-MPRAGE using FSL’s boundary based registration algorithm (epi_reg) and then warped to MNI space using the MPRAGE to MNI transformation warp images. The MNI transformed EPI images were then spatially smoothed using a 4 mm FWHM Gaussian kernel within a mask that excluded the lateral ventricles and edge voxels to minimize smoothing into cerebrospinal fluid (CSF) filled spaces. The smoothed BOLD time course was scaled with respect to the initial 12 s active baseline condition (repeating “REST”) to obtain task-induced relative % BOLD change, censored for head motion (>0.3 mm), followed by a deconvolution (AFNI’s 3dDeconvolve using 13 TENT functions between −4 and 44 s) to estimate the HRF of the 8-trial block. The HRF length was estimated to require 48 s to return to baseline by using a Matlab simulation of this study’s task design in conjunction with a biphasic impulse response function derived from a previous overt language task fMRI study (Wierenga et al., 2008). In order to account for low frequency scanner drifts, we employed the polynomial fitting option within AFNI’s 3dDeconvolve command. To quantify the dynamic evolution of the BOLD fMRI signal, the HRF was then divided into 3 segments (see Figure 2B) – the first segment defined for first 16 s (Seg1 = first 4 words), the second segment from 17th through 32 s (Seg2 = last 4 words) and 3rd segment defined from 33rd to 44th seconds for post-stimulus BOLD activity (Seg3 = post-stimulus BOLD). On each individual subject, the voxel-wise area under curve (AUC) for a given segment of the HRF was estimated and then z-transformed (see Supplementary Section S5) for subsequent secondary group analyses [Z(AUC)].

#### CBF Pre-processing

The pseudo Continuous Arterial Spin Labeling (pCASL) time course was corrected for bulk head motion using AFNI with the proton density (M0) image designated as the base. The corresponding output motion parameter file was used to compute motion parameter derivatives and flagged as a volume to censor if greater than 0.5 mm. Due to the interleaved control and label image acquisition, if a volume was flagged for censoring its corresponding label or control volume pair was also censored. A maximum of 25% of label/control pairs were allowed to be discarded due to motion to ensure data stability. The pCASL time course was then spatially smoothed with a Gaussian kernel of FWHM = 6 mm, followed by pairwise subtraction of control and label images which were averaged to generate the mean perfusion image. The signal was converted to CBF in physiological units (mL/100 g/min) by dividing perfusion image with smoothed proton density (M0) image and applying a single compartment model (Buxton et al., 1998). Finally, the CBF map was spatially transformed to MNI atlas space using a boundary-based registration (calculated via M0) to the T1w-MPRAGE in conjunction with the non-linear T1 to MNI transformation, and resampled to the task fMRI voxel size (2.2 × 2.2 × 2 mm^3^) in preparation for the sensitization methodology described next.

#### Correction Methodology to Account for Baseline CBF Variability

**FIGURE 1:**
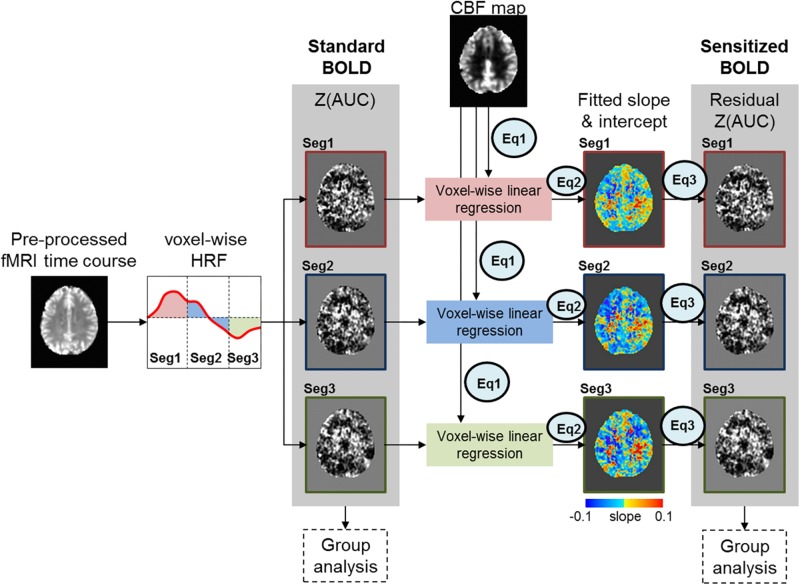
Figure above depicts the sensitization methodology to account for CBF variability embedded in task fMRI activity. The red, blue and green box outlines depict the z-transformed area under the curve [Z(AUC)] for segments 1, 2, and 3 respectively.

Figure 1 depicts our novel BOLD-correction methodology that utilizes CBF images to sensitize the task fMRI BOLD activity. The Z(AUC) maps for each segment (Figure 1: Seg1 = red, Seg2 = blue, Seg3 = green) were extracted for each participant, and denoted as ‘Standard BOLD’ in Figure 1. To ensure overlapping brain coverage across both task fMRI and CBF data, the Z(AUC) and CBF data were multiplied with a junction mask of the Z(AUC) coverage and the CBF coverage that excluded cerebellum due to the limitation from ASL coverage. The sensitization is accomplished by co-varying out the voxel-wise age-related variability in CBF from the ‘standard’ BOLD fMRI derived Z(AUC). First, the coefficients (A = intercept, B = slope) describing the CBF: Z(AUC) relationship across the group is computed using a voxel-wise linear regression (Equation 1):

(1) $$Z\,{\left(A\,U\,C\right)}_{m\,e\,a\,s\,u\,r\,e\,d,i}^{j}=A_{i}+B_{i}\cdot C\,B\,F_{m\,e\,a\,s\,u\,r\,e\,d}^{j}+\varepsilon_{i}^{j}$$

The *Z(AUC)^*j*^_*measured,i*_* denotes the Z(AUC) that was estimated from the task fMRI HRF for the *j*th voxel from segment *i*. The *CBF*_*measured*_ denotes the measured CBF value from the same *j*th voxel. The ε represents the error term or the residual. *A*_*i*_ and *B*_*i*_ denote the intercept and slope estimated for the *j*th voxel from segment *i* using the generalized Levenberg-Marquardt non-linear least squares algorithm as implemented in MATLAB (nlinfit) where we implemented a linear model function that was called within nlinfit.

(2) $$Z\,{\left(A\,U\,C\right)}_{p\,r\,e\,d\,i\,c\,t\,e\,d,i}^{j}={\hat{A}}_{i}+{\hat{B}}_{i}\cdot C\,B\,F_{m\,e\,a\,s\,u\,r\,e\,d}^{j}$$

Finally, the sensitized task-BOLD activity for *j*^*th*^ voxel and segment *i* is comprised of Z(AUC) residuals with the variance due to baseline CBF removed (Equation 3).

$$Z\,{\left(A\,U\,C\right)}_{r\,e\,s\,i\,d\,u\,a\,l,i}^{j}=$$

(3) $$Z\,{\left(A\,U\,C\right)}_{m\,e\,a\,s\,u\,r\,e\,d,i}^{j}-\left({\hat{A}}_{i}+{\hat{B}}_{i}\cdot C\,B\,F_{m\,e\,a\,s\,u\,r\,e\,d}^{j}\right)$$

Here the *Z(AUC)^*j*^_*residual,i*_* is the remaining task fMRI signal (i.e., “sensitized BOLD” in Figure 1) for *j*th voxel in segment *i* that more accurately estimates the task-induced BOLD activity after accounting for the voxel specific (i.e., brain area) age-related variability in baseline CBF signal (*CBF*_*measured*_). ${\hat{A}}_{i}$ and ${\hat{B}}_{i}$ are the estimated intercept and slope for the *i*th segment respectively.

In order to investigate the achieved power with 24 subjects, we utilized linear bivariate regression approach where achieved power was investigated using the slope from Equation (1) and standard deviations for Z(AUC) and CBF across the 24 subjects for a given brain area.

#### Age Dependency of CBF and Its Effect on BOLD Sensitization

Given that CBF has a strong dependency on age (Lu et al., 2011), from a methodological standpoint, it is imperative to check whether the age variability encoded within the CBF need to be retained or removed prior to BOLD sensitization. For simplicity, we denote age retained CBF value as *CBF_*measured*_*, and age removed CBF value as *CBF*_*age–corrected*_. To determine if age should be removed from the CBF values prior to sensitization, we conducted the BOLD-correction steps described in Figure 1 using either the *CBF*_*measured*_ or *CBF*_*age–corrected*_. The age-corrected CBF values and subsequent “un-sensitized” Z(AUC) values were obtained as described in Supplementary Section S2. To test the effect of correcting age in the CBF values prior to sensitization, we employed point bi-serial correlation to investigate the young versus old age dependence of *Z(AUC)_*standard*_*, *Z(AUC)_*residual*_* (i.e., “sensitized”), and *Z(AUC)_*un–*__*sensitized*_* and reported the *R*^2^ and *t*-value obtained via point bi-serial correlation analysis.

#### Leave-One-Out Cross Validation

In order to examine the stability of our proposed group-level normalization (described in the previous section Correction Methodology to Account for Baseline CBF Variability), we employed leave-one-out (LOO) cross validation (CV) approach wherein we leave out one individual (say subject-1) and fit the proposed model using the remaining individuals (i.e., subjects 2 through 24). We then use this fitted model to predict the Z(AUC) for subject-1 and then compute the prediction error for that subject as cve1 = (ZAUC_*obs*__1− ZAUC_*pred*__1)^2^, where ZAUC_*obs*_ is the observed Z(AUC) and ZAUC_*pred*_ is the predicted Z(AUC). Then we repeat this process for all the 24 subjects and obtain [cve1,cve2,.,cve24]. The cross validation error is computed as the mean of [cve1,cve2,.,cve24]. The % error for a given subject-1 is calculated as PE_1 = 100^∗^(ZAUC_*obs*__1 – ZAUC_*pred*__1)/ZAUC_*obs*__1. Finally, we computed the cross validation % error as the mean of [PE_1, PE_2,., PE_24]. We implemented the leave-one-out CV approach to estimate the stability for estimated ‘*B*_*i*_’ coefficient in Equation (1), and subsequently the average % error was estimated for areas found to have improved Brain-behavior relationships.

The LOO CV approach was also utilized to test whether the proposed sensitization methodology resulted in improved prediction of behavior compared to standard BOLD (no correction) or normalization by division with CBF (see section Voxel-Wise Division by CBF Normalization on Individual Subject Below). Our approach was as follows: (a) obtain the R^2^_*sensitized*_ and R^2^_*standard*_ values for N-1 subjects, N times, (b) Fisher-z transform the distribution of R^2^ values, and (c) test the significance between the standard and sensitized Fisher-z transformed R^2^ values using a paired t-test. In addition to predictability of behavior, the LOO CV was also utilized to estimate the stability in predicting behavior across the methodologies.

#### Group Analyses

Significant group differences in the *CBF*_*measured*_ were determined via AFNI’s 3dttest++ and 3dclustsim (*p* < 0.01, FWE-corrected cluster size = 1646). Within group and between group analyses were carried out on *Z(AUC)_*standard*_* and sensitized *Z(AUC)_*residual*_*. Familywise error (FWE) corrected cluster-size thresholds were obtained through Monte Carlo (MC) simulation which estimates the spatial correlation of voxels, cluster detection thresholds, and cluster identification (Cox et al., 2017) as implemented in the *ClustSim* program of the latest version of AFNI. Such an approach provides an estimated cluster size at a desired voxel-wise alpha threshold (*p* < 0.01) and false positive rate (FPR) < 5%. Significant areas of activation for the overt category member generation task were identified for each segment, and each group.

To investigate the effect of sensitization on BOLD-behavior relationships, linear regression was carried out between in-scanner behavioral measures (i.e., % accuracy of correct semantic item) and average *Z(AUC)_*standard*_* or *Z(AUC)_*residual*_*. The average *Z(AUC)_*standard*_* or *Z(AUC)_*residual*_* for each participant was calculated by averaging the Z(AUC) value across all voxels within a significant group-level area of activation (*p* < 0.01, FWE corrected). Then group level linear regression for each segment *i* was carried out between cluster-averaged *Z(AUC)_*standard,i*_* or *Z(AUC)_*residual,i*_* and behavior *(%accuracy_*i*_*), and corrected for multiple-comparison using Bonferroni correction (i.e., p_*corr*_ = 0.01/number of significant brain areas). In addition, the effect size (f^2^) was calculated to explore the impact of increased R^2^ due to sensitization.

#### Voxel-Wise Division by CBF Normalization on Individual Subject

As a comparator to our proposed group-level normalization approach, we also examined voxel-wise division of BOLD activity with corresponding CBF value in each individual subject (Bandettini and Wong, 1997). Group level maps were generated for each segment, and BOLD-behavior relationships were also estimated as described in the previous section Group Analyses.

## Results

### Percent (%) Accuracy of In-Scanner Language Behavior

**FIGURE 2:**
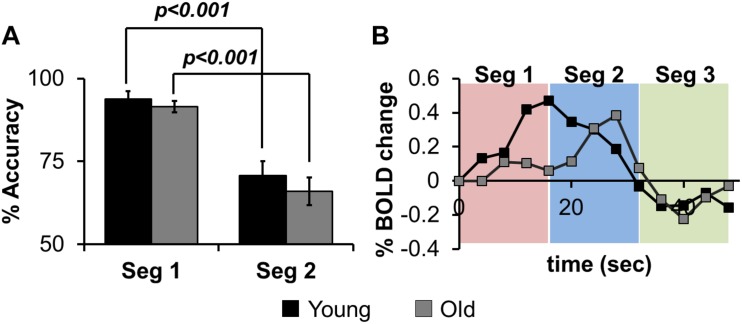
**(A)** the% accuracy of in-scanner language behavior for both groups (Young, Old) for Seg1 (first 4 words) and Seg2 (last 4 words) (see text for details). **(B)** group-averaged HRF from left Angular gyrus divided into Seg1 (first 4 words), Seg2 (last 4 words), and Seg3 (post-stimulus BOLD response).

Figure 2A shows the results for in-scanner behavior analyses for % accuracy of correct word generation. Within segments 1 and 2, there are no significant group differences, but across segments, both groups perform significantly worse (lower accuracy) in Seg2, suggesting that irrespective of age group there is a reduction in the ability to accurately retrieve exemplars as a function of task evolution. Figure 2B depicts group-averaged HRF from left angular gyrus that is significantly (*p* < 0.01) involved in the task. Visual inspection of the HRF confirms that the sparse sampled data acquisition and subsequent image processing resulted in high quality HRF across the brain that could reliably be used for quantification of Z(AUC). We also note that the z-transformation of AUC was well approximated to standard normal distribution with slight skewness (see Supplementary Section S5).

### CBF Group Differences

**FIGURE 3:**
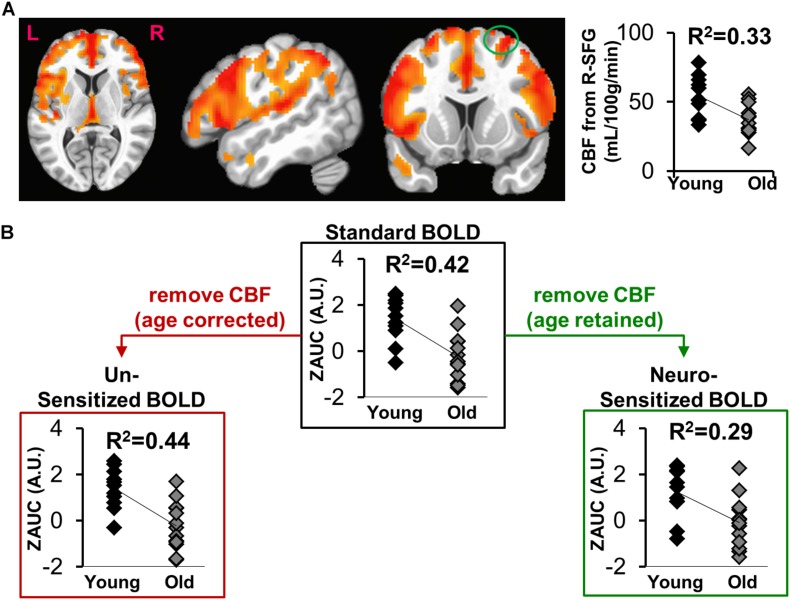
**(A)** Voxel-wise map of age-specific CBF differences (Young > Old), with the plot depicting group comparisons of CBF in right Superior Frontal Gyrus (R-SFG, denoted as green circle). **(B)** The effect of sensitization on standard BOLD activity (ZAUC, outlined by black box) quantified from R-SFG. The results in red box is where sensitization was carried using age corrected CBF, while the results in green box is where sensitization was carried out using CBF in which age was retained.

Voxel-wise CBF group differences (Young – Old) were computed (Figure 3A, *p* < 0.01, cluster size = 1646), showing age-related decrease of CBF in frontal, parietal, temporal and sub-cortical areas. Average CBF was quantified within right superior frontal gyrus (L-SFG) for each participant and compared across groups via point bi-serial correlation to show a significant aging-related decrease in CBF (*R*^2^ = 0.33, *t* = 3.26, *p* = 0.0034).

### Should the Age Encoded in CBF Be Retained for BOLD Sensitization?

Figure 3B shows the effects of sensitization on standard BOLD activity quantified from right superior frontal gyrus (R-SFG). The sensitization was carried out using *CBF*_*measured*_ (age-retained, Figure 3B green box) or *CBF*_*age–corrected*_ (age removed. Figure 3B red box). A significant age relationship is observed in *Z(AUC)_*standard*_* (*R*^2^ = 0.42, *t* = 3.91, *p* = 0.0007), which is reduced in *Z(AUC)_*residual*_* (“sensitized”)(*R*^2^ = 0.29, *t* = 2.96, *p* = 0.007) potentially due to removal of vascular age differences, thereby sensitizing the BOLD signals to neural-specific age differences. When BOLD is sensitized using age-corrected CBF (*Z(AUC)_*un–*__*sensitized*_*), the age and BOLD relationship remains similar to that of *Z(AUC)_*standard*_* (*R*^2^ = 0.44, *t* = 4.13, *p* = 0.0004), suggesting that the age effects were retained in *Z(AUC)_*un–*__*sensitized*_*. Since sensitization using *CBF*_*measured*_ was able to sensitize the BOLD activity, subsequent results are based on sensitization using age-retained CBF.

### Effectiveness of Task-BOLD Sensitization

**FIGURE 4:**
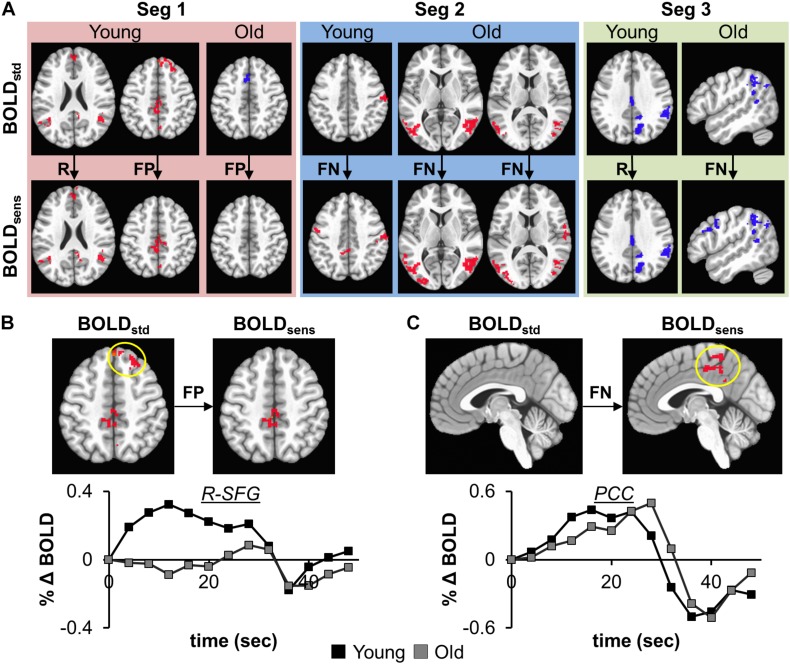
**(A)** depicts the effectiveness of task-BOLD sensitization using age-retained CBF for each segment (Seg 1–3). The effectiveness of sensitization is characterized based on removal of false positives (FP), identification of false negatives (FN), and retaining (R) expected activity. Red depicts increased BOLD activity while the blue depicts decreased BOLD with respect to the baseline BOLD activity. The bottom panels show the cluster-averaged hemodynamic response function quantified from standard BOLD activity in right superior frontal gyrus (**B**, FP) and posterior cingulate (**C**, FN) in young participants (*p* < 0.01, FWE corrected). Yellow circle denotes the brain area. The axial images are in neurological convention. BOLD_*std*_ refers to Z(AUC)_*standard*_ and BOLD_*sens*_ refers to Z(AUC)_*residual*_ in the methods section.

The effectiveness of task-BOLD sensitization was characterized based on removal of false positives (FP), identification of false negatives (FN), and retaining (R) expected task-specific activity after group analyses (both within group and between-group, *p* < 0.01, FWE corrected). For between-group analyses, we identified removal of FP activity from R-SFG in Seg1. No other significant between-group differences were identified for other segments. Effective sensitization was observed within group in all three segments (Figure 4A and Table 2), and was effective for both BOLD activation and deactivation in both age groups. False positive activity was observed only in Seg1, and not Segments 2 and 3. The recovery of false negative activity was observed in only Segments 2 and 3. The retained activity which was left unchanged by sensitization was observed in all three segments.

**TABLE 2:** Significant (voxel-wise p < 0.01, FWE corrected) within-group BOLD activity for each segment.

	**Seg 1**					**Seg 2**					**Seg 3**				
	**Area**	**MNI (peak)**	**Grp**	**Cluster size (mm^3^)**	**BOLD activity**	**Area**	**MNI (peak)**	**Grp**	**Cluster size (mm^3^)**	**BOLD activity**	**Area**	**MNI (peak)**	**Grp**	**Cluster size (mm^3^)**	**BOLD activity**
**FP**	R-SFG	24, 33, 48	Y	1955	+	*None*					*None*				

	L-preSMA	−9, 13, 50	O	1297	−										
**FN**	*None*					PCC	−2, −22, 46	Y	3030	+	R-PMd/R-Pop	40, 4, 40	O	2294	−
						L-PMd/L-POp	−55, −5, 44	Y	1868	+					
						L-M1	−13, −18, 76	Y	1549	+					
						R-STG	64, −33, 22	O	1781	+					

**R**	PCC/PCUN	−2, −27, 46	Y	6369	+	R-postCent	59, −20, 48	Y	7289	+	R-IPL/R-MTG	59, −42, 34	Y	10319	−
	R-Ling/R-V1	11, −88, 4	Y	2536	−	PCUN/PCC/R-M1	−4, −47, 34	O	33125	+	R-PCUN	15, −64, 30	Y	5643	−
	L-Ling/L-V1	−9, −91, −2	Y	2478	−	R-MOG	51, −69, 6	O	6476	+	PCC	2, −25, 28	Y	4317	−
	mPFC (dorsal)	0, 50, 24	Y	2207	+	L-MOG	−48, −73, 0	O	5101	+	R-IPL	42, −51, 50	O	10871	−
	mPFC (ventral)	9, 57, 10	Y	1859	+	L-postCent/L-M1	−51, −36, 56	O	1946	+	PCC	2, −36, 40	O	4133	−
	R-AG	46, −53, 24	Y	1771	+						bilateral PCUN	−11, −69, 28	O	3930	−
	L-AG	−57, −62, 20	Y	1210	+										

**The false positive (FP), false negative (FN) and retained (R) BOLD activity were identified by comparing the sensitized group results to that from standard BOLD analysis. The “+” and “−” sign denoted the BOLD activation and deactivation with respect to resting BOLD activity; L- and R- prefixes denotes left and right hemisphere brain area; Y, young; O, old; SFG, superior frontal gyrus; pre-SMA, anterior supplementary motor area; PCC, posterior cingulate area; PCUN, precuneus; Ling, lingual gyrus; V1, primary visual cortex; mPFC, medial pre-frontal cortex; AG, angular gyrus; PMd, dorsal pre-motor cortex; Pop, pars opercularis; M1, primary motor cortex; STG, superior temporal gyrus; postCent, post-central gyrus; MOG, middle occipital gyrus; IPL, inferior parietal lobe; MTG, middle temporal gyrus*.*

To ascertain that the FP activity in R-SFG from standard BOLD analysis was not due to any artifacts (such as motion), we quantified the underlying HRF (see Figure 4B) and did not find any uncharacteristic HRF features. To ensure that the detection of R-SFG as a FP area was not due to a statistical thresholding issue, we computed voxel-level pairwise difference (i.e., ZAUC_*standard*_ – ZAUC_*sensitized*_) maps (*p* < 0.01, FWE corrected) and found that the R-SFG area was non-zero, indicating that covarying out baseline CBF indeed changed the activation profile rather than simply reducing the detection power. Consistent results were also observed for the data from the other false positive segments including FP areas in the Older group. Further, to ensure that the FN activity that was recovered after sensitization stems from an HRF evolving like a BOLD response, and not like a statistical artifact, the HRF from each FN area were visually inspected across both groups. For example, the posterior cingulate % BOLD change estimated from standard BOLD analysis (see Figure 4C) indeed evolves like a classic HRF, but was not detectable before sensitization, thereby demonstrating the effectiveness of our sensitization approach.

We also applied the sensitization approach on Z(AUC) quantified from the entire HRF (i.e., without any segmentation). In young cohort, sensitization resulted in FN activity in right postcentral gyrus (MNI peak co-ordinates: 40, −33, 64; activated cluster size = 1694 mm^3^), and in the old cohort, sensitization resulted in retained activity in right superior occipital gyrus (MNI peak co-ordinates: 28, −59, 36; activated cluster size = 2410 mm^3^) suggesting that our methodology is effective even on Z(AUC) quantified from entire HRF, and that the segmentation is more dependent on the experimental design and scientific question.

### Sensitization and BOLD: Behavior Relationships

In this study, we hypothesized that sensitization of BOLD signal should enhance its relationship with behavioral measures. To test this hypothesis, we carried out linear regression between BOLD (*Z(AUC)*) and behavior (i.e., % accuracy in correct responses) for both standard and sensitized approaches. As seen in Table 3, for a very conservative Bonferroni corrected threshold (i.e., p_*corr*_ = 0.01/3 = 0.003), we do not observe any significant BOLD-behavior relationship. However, marginally significant relationship (p_*corr*_ = 0.1/3 = 0.03) was observed between *BOLD*_*sens*_ from left dorsal pre-motor cortex (PMd) and behavior in the young group. Lesser marginally significant relationships were observed for right post-central gyrus (*R*^2^ = 0.30; *p* = 0.08) and precuneus + posterior cingulate (PCUN/PCC) complex (*R*^2^ = 0.12; *p* = 0.09) after sensitization. As shown in Supplementary Figure S8 (Supplementary Section S6), the mean estimates of Fisher z-transformed *R*^2^ for the proposed sensitized methodology were larger than the standard approach, and the normalization by division approach had even lower mean estimates compared to the other two methods. Paired t-test showed significant difference (*p* ≤ 0.0001) between the methodologies across all three brain areas. On the other hand, the stability in prediction of behavior across the 3 methodologies showed a CV error range of −20 to 20 (see box plots in Supplementary Section S7), and were not statistically different.

**TABLE 3:** Brain areas that showed Bonferroni corrected significant relationship (R^2^) between task activity and behavior.

**Area**	**Group**	**N**	**Seg**	**BOLD_*std*_*R*^2^ (p_*corr*_)**	**BOLD_*sens*_*R*^2^ (p_*corr*_)**	**Increase in *R*^2^**	**Effect size (f^2^)**
L-PMd	Y	11	2	0.35 (0.06)	0.48 (0.02)^§^	0.13	0.25
R-postCent	Y	11	2	0.28 (0.09)	0.30 (0.08)	0.02	0.03
PCUN/PCC	Y&O	24	2	0.06 (0.25)	0.12 (0.09)	0.06	0.07

**N, number of participants recruited for the study; Seg, segment; std, standard; sens, sensitized; Y, young; O, old; L-PMd, left dorsal pre-motor area; R-postCent, right post-central gyrus; PCUN, precuneus; PCC, posterior cingulate; p_*corr*_, Bonferoni corrected p-value; ^§^ marginally significant at p_*corr*_ = 0.03; the effect size is estimated for an increase in R^2^ and the desired statistical significance was set for p_*corr*_ = 0.003.**

For the brain areas that were found to show increased BOLD-behavior relationships in Table 3, the achieved power for a critical | t| = -2.07 are: 56.67% (L-PMd), 11.04% (R-postCent) and 56.19% (PCUN/PCC). In terms of the stability of the proposed methodology, from the leave-one-out CV analysis we observed an average % error of 33.7% (L-PMd), 52.6% (R-postCent), and 30.4% (PCUN/PCC), and the CV error (mean ± std. error) of 0.0026 ± 0.17 (L-PMd), −0.0025 ± 0.17 (R-postCent), and 0.0064 ± 0.17 respectively. The effect size associated with increase in R^2^ due to sensitization was small to intermediate. Supplementary Table S2 summarizes the age effects on BOLD activity (standard and sensitized) and CBF values quantified from the above mentioned areas that had improvements in BOLD-behavior relationship due to sensitization.

To verify that the contribution to behavior is primarily from task-specific neural substrates and not baseline CBF, linear regression was carried out between CBF and behavior, resulting in non-significant relationships: L-PMd (*R*^2^ = 0.02, *p* = 0.7); R-postCent (*R*^2^ = 0.01, *p* = 0.77); PCUN/PCC (*R*^2^ = 0.03, *p* = 0.43). Since CBF does not relate with behavior, the vascular component did not account for any changes in BOLD-behavior relationships. As shown in Table 3, our results show False-Negative (FN) areas (such as L-PMd and PCC in Young), and Retained (R) areas (such as R-postCent in Young) that showed improved BOLD-behavior relationship due to sensitization. Further, it is also imperative to investigate how the False-Positive (FP) areas shown in Table 2 relate with behavior. For R-SFG (which is a FP area in Young), the BOLD-behavior relationship is: *R*^2^ = 0.15 (p_*uncorr*_ = 0.24) for ZAUC_*standard*_ and *R*^2^ = 0.17 (p_*uncorr*_ = 0.21) for ZAUC_*sensitized*_. For L-preSMA (which is a FP area in Old), the BOLD-behavior relationship is: *R*^2^ = 0.01 for both ZAUC_*standard*_ and ZAUC_*sensitized*_. Indeed, it is promising to note that these areas (identified as FP) do not have a meaningful relationship with the behavior while the proposed technique still sensitized the R-SFG ‘standard’ BOLD activity. These results support the premise that sensitization of task-BOLD signal enhances the BOLD relationship (from task-specific false-negative and retained areas) with the behavior.

### Sensitized Maps of BOLD Activity

**FIGURE 5:**
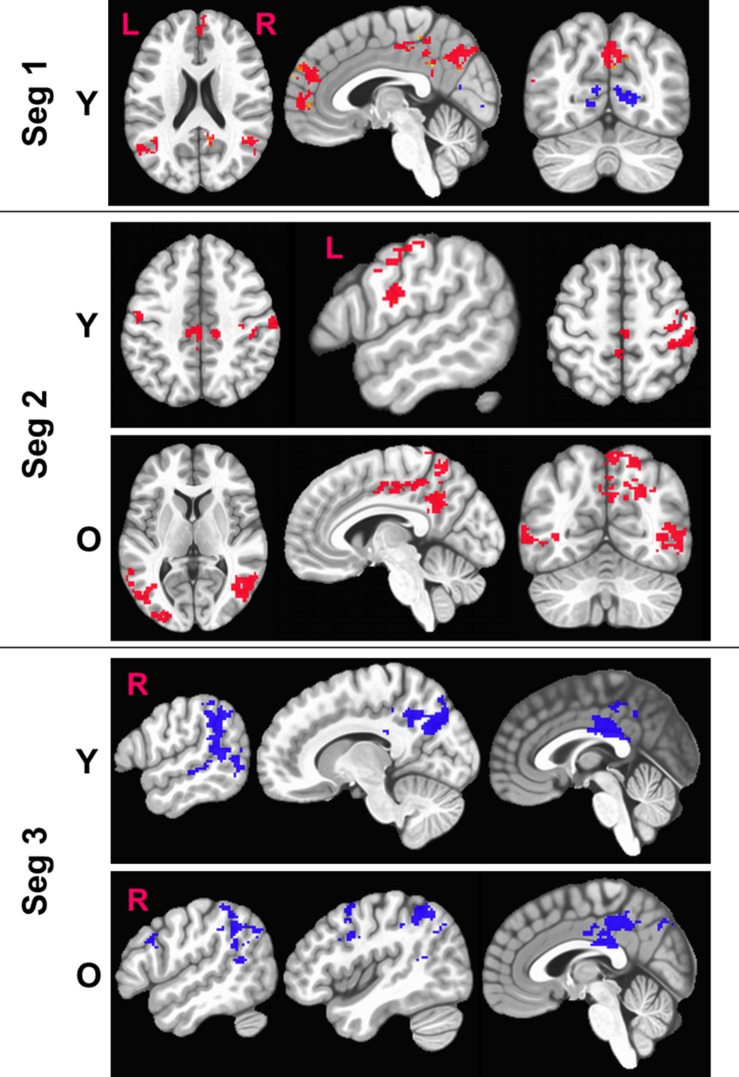
Figure above shows within-group significant (voxel-wise *p* < 0.01, FWE corrected) maps of z-transformed BOLD (sensitized) activity. The BOLD activity is quantified separately for each segment (Seg). Red denotes BOLD activation while blue denotes BOLD deactivation with respect to baseline. L, Left; R, right hemisphere.

Figure 5 shows the sensitized group maps for task-specific BOLD activity. For Seg1, we observed significant activation (voxel-wise *p* < 0.01, FWE corrected cluster size = 119, false positive rate (FPR) < 5%) only in the young group. Specifically, BOLD activation was observed in the default mode areas (anterior cingulate, medial pre-frontal cortex, bilateral angular gyrus, and posterior cingulate) and BOLD deactivation in bilateral lingual gyri and primary visual cortex areas. No significant activity was observed within the old group or between the young and old groups. For Seg2, we observed significant (voxel-wise *p* < 0.01, FWE corrected, cluster size = 158, FPR < 5%) in both young and old groups. In younger participants, we observed BOLD activation bilaterally in motor cortices, dorsal premotor (PMd) cortex, and pars opercularis (POp) s. On the other hand, in older participants, delayed BOLD activation was observed in posterior default mode areas (posterior cingulate and precuneus), and bilaterally in the superior and middle temporal gyri. Interestingly, this indicates that the older group significantly engaged (or failed to disengage) the DMN in Seg2 relative to the control condition, while the younger group engaged these areas in Seg1. For Seg3, significant (*p* < 0.01, FWE corrected, cluster size = 188, FPR < 5%) BOLD deactivation during the post-stimulus phase was observed in both groups, including deactivation in posterior perisylvian areas, and posterior cingulate. In addition, the older group deactivated right POp and PMd. There were no significant between group differences (*p* < 0.01, FWE corrected, FPR < 5%) for sensitized BOLD activity either for entire HRF or for segmented HRF.

### Normalization by Division Approach

Figures in Supplementary Section S4 summarizes the within-group maps (*p* < 0.01, FWE corrected, FPR < 5%) for normalization by division (i.e., CBF) approach for each segment and how these results compare with the standard and the proposed group-level normalization by covariate approach. Visual inspection of the resulting maps are more or less similar to “standard” BOLD maps wherein false positives were retained (solid brown circles in Supplementary Figures S1–S6), true positives were not identified, and in fact, the normalization by division approach removed key task-specific areas (dashed yellow circles in Supplementary Figures S1–S6). We also estimated the BOLD-behavior relationships from the same areas as in Table 3 for the normalization by division methodology and obtained the following *R*^2^: 0.20 (L-PMd), 0.21 (R-postCent), and 0.07 (PCUN/PCC), which describe less variance than our proposed methodology and do not reach significance.

## Discussion

The main objective of this study was to develop a multimodal approach that allows for co-varying out the baseline CBF such that task fMRI (e.g., language) signals are sensitized to more accurately reflect age-specific neural activity differences. This study is novel in several aspects. First, we have developed a methodology that combines task fMRI and ASL measures to sensitize BOLD activity by removing voxel-wise age-related variability encoded in baseline CBF. Second, analyzing the BOLD dynamics and behavior as a function of within-block task evolution (i.e., language processing and speech production) allowed for an improved understanding of the neurobiology of aging associated with word retrieval as measured by overt category generation. Finally, our data acquisition design and approach probed into the domain-general aspects of word finding and word production in a semantically-driven verbal fluency task.

### Sensitization of Task-BOLD Activity

BOLD is a complex neurovascular signal that is modulated by local variations in CBF, cerebral blood volume, and cerebral metabolic rate of oxygen consumption (CMRO2) induced by neuronal activation or vascular challenges (Buxton, 2012). Depending on the complex interplay of these variables, the dynamics of BOLD activity may not reflect solely the underlying change in neural activity, especially when comparing populations (such as younger versus older adults) with different neurovascular characteristics (Liu, 2013). Increased inter-subject variability in baseline physiologic variables (such as CBF) due to non-neural factors leads to significant variability in BOLD amplitude (D’Esposito et al., 2003) and removal of such systematic variance (i.e., baseline CBF) from task BOLD activity improved the detectability of brain activation.

Figure 1 and Equations (1) through (3) describes our multi-step linear regression approach to remove the variability in baseline CBF that is encoded in task-induced BOLD activity. We strategically combined task fMRI and ASL images (see Figure 1) that were acquired during the same scanning session. Considering the CBF group difference maps (Figure 3A), we observed an age-related decline in frontal, temporal and parietal areas as expected (Lu et al., 2011). In some of those critical brain areas (such as frontal regions), we also observed significant age-related task fMRI differences using the “standard’ analysis. Given that both BOLD and CBF have age dependence, the question is how much of the age-specific between-group BOLD difference can be attributed to neural activity. As shown in Figure 3B, for “standard” BOLD analysis, there is a strong association (*r* = 0.65) between *Z(AUC)_*standard*_* and age. By applying our sensitization approach, the association between sensitized BOLD (*Z(AUC)_*residual*_*) and age dropped to *r* = 0.54 potentially due to removal of between-group variability in baseline CBF. However, when age was first removed from CBF and then the age-corrected CBF was regressed out from “standard” BOLD, the association between “unsensitized” BOLD (*Z(AUC)_*unsensitized*_*) and age (*r* = 0.66) was not different from “standard” BOLD implying that using age-corrected CBF for sensitization does not remove between-group variability in baseline CBF. Therefore, the variability in baseline CBF is highly age-dependent, such that aging-related changes must be retained in CBF to sensitize the task-BOLD to age-related neural differences. The proposed sensitization approach which regresses out age-retained CBF from BOLD minimizes baseline physiologic age-related differences, thereby enhancing the BOLD sensitivity to task-specific neural-related age differences.

Brain function, and hence behavior is the result of underlying neural activity, suggesting that the process of BOLD sensitization should improve BOLD-behavior relationships. In this study we quantified accuracy of generating correct responses inside the scanner while the participants were engaged in the task with the intent of relating the behavior to Z(AUC). As shown in Table 3, we observe that language and speech relevant areas show higher correlation with % accuracy after sensitization. It was promising to observe that the correlation between L-PMd activity and % accuracy not only increased (standard: *r* = 0.59; sensitized: *r* = 0.7) but was also marginally significant (*p* = 0.02) only after sensitization. Because this task involves overt word generation, the role of left dorsal pre-motor cortex (L-PMd) is relevant as there is evidence that L-PMd along with left pars opercularis (L-POp) is involved in sound sequencing and assembly (Twomey et al., 2015). We also observed a strong (*r* = 0.55) but less marginally significant (*p* = 0.08) correlation between sensitized posterior DMN activity and % accuracy. There is growing evidence that each sub-region in the default mode network (DMN) may sustain a specific functional role in cognitive processing (Beckmann et al., 2009; Laird et al., 2011), and further, functional heterogeneity across core DMN nodes have been reported in semantic processing and speech production tasks (Seghier and Price, 2012). Interestingly, baseline CBF quantified from the above-mentioned specific areas did not have any significant relationship with behavior which implies that the increase in association between task-BOLD and behavior is due to task-specific, behaviorally driven neural activity as indicated by sensitized (but not standard) BOLD. From Supplementary Table S2 we note that the aging-related decline in CBF in these areas is consistent with previous literature and that the proposed sensitization approach reflects more accurate age-specific decline in BOLD activity resulting in more meaningful sensitized BOLD-behavior relationships. The effect of sensitization on the increase in association between Z(AUC) and behavior is moderately high (*f*^2^ = 0.25) in language/speech related areas such as L-PMd (see Table 3). Further, adequate power is achieved to sensitize the task-BOLD activity with 24 subjects, but varies across brain areas possibly because different brain areas have differences in underlying physiology wherein some areas (i.e. R-postCent) require > 100 subjects to achieve adequate power while for L-PMd and PCUN/PCC, our data suggests that an additional 16 subjects would result in desired power of 0.8. Nevertheless, given the pilot nature of this study, it is encouraging to note that our sensitization approach can result in an improved association between sensitized task-BOLD and behavior, whereas a weak relationship existed when BOLD responses were not sensitized.

From a methodological standpoint, we also investigated whether our sensitization approach resulted in enhancing the task-specificity (i.e. removal of false positives, FP) and task-sensitivity (i.e. extracting false negatives, FN). For removal of FP, one striking area that was significant in “standard” BOLD analysis but not after sensitization is the right superior frontal gyrus (R-SFG) in the young group. Given that fMRI lateralizes language processing primarily to the left hemisphere (Frost et al., 1999), particularly in younger participants (Wierenga et al., 2008), significant (p < 0.01, FWE corrected, < 5% FPR) activation in R-SFG resulting from standard BOLD analysis was debatable. Further, the baseline CBF from this area in younger group was within normal range which should not have inadvertently affected the resulting task-induced BOLD activity. However, after sensitization and at the same significance threshold, R-SFG activity from “standard” BOLD was identified as a FP error. Although, SFG activity can be involved in executive control functions (Solbakk et al., 2008), in the context of task-sensitization and given that language is left-lateralized, R-SFG activity identified in “standard” BOLD analysis is indeed FP that can lead to misinterpretation if not sensitized. Also, given that these FP areas did not relate with behavior, and were not an artifact of statistical thresholding at fMRI group level analyses, it is promising to note that sensitization of task-BOLD signal is indeed important. On the other hand, identification of FN is also equally important. From Table 2, we note that L-PMd/L-POp activity is significant (p < 0.01, FWE corrected, < 5% FPR) and detectable only after sensitization. As explained above, L-PMd and L-POp are speech and language eloquent areas that are involved in sound sequencing and phonological processing (Twomey et al., 2015) respectively. Similarly, involvement of anterior posterior cingulate cortex (PCC) was detectable only after sensitization. Based on previous evidence that anterior PCC is involved in semantic processing (Seghier and Price, 2012), we hypothesize that it is involved in the attentional aspects of stimulus-specific semantic processing. In addition, we investigated whether our sensitization approach negatively impacted the task activity from expected areas (i.e. areas that are neither FP nor FN and we denote these areas as ‘retained’ in Table 2). We observed that several of the classic language eloquent areas (see Table 2) remained unaffected after sensitization. For the results in Table 3, while it is reasonable to expect Z(AUC)_*standard*_ to also potentially reach significance in describing the explained variance (R^2^) in the behavior, the key message of this study is that physiological variability encoded in BOLD induces false positives and masks true positives which need to be corrected for stand-alone accurate interpretation of task fMRI results (i.e. even in the absence of behavioral data). Also, the sensitized data are essentially derived from the standard BOLD data which means that they are inherently not independent of each other to act as ‘unique’ predictors. Although multiple linear regression modeling (for simultaneously incorporating both ZAUC_*standard*_ and ZAUC_*sensitized*_ to predict Behavior) can be a potential approach in exploring how each of the methods predict unique variance in the behavior, at this point it is unclear whether such an approach adds clarity in accurately characterizing the underlying sensitization. As shown in Supplementary Section S8, using the multiple linear regression (MLR) approach, the behavior is mischaracterized with 2 ^∗^ task-induced neural activity which is not physiologically meaningful, and further the MLR approach incorporates the baseline vascular component (i.e., V) which does not relate with behavior as described above. On the other hand, we observe that the predictability of behavior using our simple sensitization approach is significantly different from the standard approach (see Supplementary Figure S8). Collectively, these results suggest that our proposed sensitization method is effective in improving the accuracy of task fMRI maps.

From Figure 5, we also observe that the FPs were primarily observed in Seg1 and FNs in Seg2. Although our current techniques did not allow for direct measurement of coupling between BOLD and CBF, we observed consistent inverse relationship between task-BOLD Z(AUC) and CBF as observed in previous studies (Lu et al., 2008; Liau and Liu, 2009). Based on associative relationship between task-BOLD and CBF from this study, our results suggest that FPs identified in Seg1 of “standard” BOLD analysis could be due to variability in the baseline physiology (i.e. transient fluctuations in BOLD-CBF coupling) during the early stages (i.e. Seg1) of task evolution that may potentially lead to variable BOLD activity. However, by the time the task has evolved to Seg2, the BOLD-CBF coupling may have stabilized such that FNs may become more detectable after sensitization.

Normalization by division on an individual subject level is a widely employed approach to sensitize task fMRI signals (Bandettini and Wong, 1997; Liu et al., 2013). However, systematic experimental and theoretic work from Liau and Liu (Liau and Liu, 2009) have shown that voxel-wise normalization based on division (i.e. voxel-wise BOLD activity divided by corresponding CVR) resulted in increased inter-subject variability. By employing a rigorous analytic procedure Liau and Liu showed that the normalization by division approach does not accurately account for the intercept in the linear relationship between task BOLD and physiologic measure (which in their case was hypercapnic BOLD response), and thereby increased inter-subject variability. As such, in our study we are attempting to correct for age-related variability in baseline CBF, and when we approach this problem using the individual subject voxel-wise normalization by division (i.e. CBF), we found that – (i) visual inspection of the resulting maps are more or less similar to ‘standard’ BOLD maps wherein false positives were retained, true positives were not identified, and in fact, the normalization by division approach removed some key task-specific areas (see figures in Supplementary Section S4), and (ii) The predictability of behavior using voxel-wise normalization by division approach was significantly weaker compared to the proposed methodology. Therefore, based on this evidence we firmly believe that our proposed methodology is in agreement with other rigorous theoretic work (Liau and Liu, 2009) wherein co-varying out the physiologic measure (i.e. CBF) from task BOLD is more accurate than normalization by division approach.

### BOLD Dynamics as a Function of Within-Block Task and Functional Evolution

Segmenting the BOLD HRF helped improve our understanding of language processing in several ways. First, as shown in Figure 5, we observe delayed task activity in the older group. Secondly, from Table 2 we observe that FP primarily occurs in Seg1 and FN are detected in segments 2 and 3. Identification of FPs and FNs and thereby exploiting the limitations of “standard” BOLD analysis was facilitated by segmenting the BOLD dynamics as a function of task evolution. Thirdly, typical block analysis constitutes estimating the task betas for the designed block length, and such an approach would have negated the sensitivity to task-driven BOLD activation and deactivation. However, our approach of quantifying the HRF followed by the estimation of area under the curve for each segment allowed for a more granular investigation of the task-induced BOLD dynamics (i.e., functional evolution) as a function of serially presented task stimuli (i.e., task evolution). Further, it should be noted that from a pure sensitization standpoint, our proposed methodology worked even on non-segmented HRF. This underscores the point that sensitization is required for improving the accuracy of BOLD activity while the segmentation of BOLD activity allowed for a better understanding for aging-related BOLD dynamics and BOLD-behavior relationships (for language task) as described below.

### Aging-Related Neurophysiological and Cognitive Findings

As shown in Figure 5, the younger group activates attentional components of DMN in Seg1 as they are potentially engaging in the task immediately, while the older group shows such an activity in Seg2 indicating that they may be slower to engage or re-engage with the task after a period of rest. Literature suggests that aging leads to increased stiffening of vessels (Podlutsky et al., 2010; Trott et al., 2011) that can lead to increased delay in vessel compliance (Chiacchiaretta et al., 2018) which may contribute to delayed task activity in older adults. Given that task-induced change in BOLD activity also incorporates the influence of baseline CBF (Davis et al., 1998), delayed DMN activity in the older group may perhaps be due to compromised vessel compliance, especially since it has been indicated that the resting BOLD-CBF coupling is strongest within major brain networks such as the DMN (Tak et al., 2014).

In the context of semantic processing of category member generation, the dynamics of language behavior (i.e., %accuracy) and supporting brain physiology (i.e., task-BOLD activity) within a block is not well understood. To the best of our knowledge, the current work is the first to address such a gap in understanding. Irrespective of age group, % accuracy in language performance within a block drops significantly as a function of task evolution. In light of this phenomenon, we segmented the BOLD HRF into the first four category stimuli, the last four category stimuli, and the post stimulus BOLD activity. We observed biphasic BOLD activity across various task-specific areas (see Table 2 and Figure 5) irrespective of age group which is consistent with a previous overt word generation study (Wierenga et al., 2008). Due to the biphasic nature of the HRF, significant BOLD deactivation is observed across multiple brain areas in Seg3 for both young and old groups. Our task was manipulated for semantic difficulty via the categories. Specifically, the deactivation in anterior PCC could be in response to greater demands on semantic processing (Seghier and Price, 2012), specifically, introspection of semantic difficulty during the post-stimulus phase (segment 3). The biphasic BOLD response with greater BOLD deactivation has also been observed in other cognitive paradigms such as visual attention and working memory (Tomasi et al., 2006), where the magnitude of deactivation was larger for working memory tasks with graded level of difficulty. The observation of biphasic BOLD responses in our study may be due to the involvement of both visual attention (i.e., continuously attending to externally guided visual stimuli via “reading”), and verbal working memory (to accurately execute the task of producing correct exemplars without repeats). Thus, at this point it is not clear whether greater BOLD deactivation in specific DMN areas is a reflection of greater semantic demands or an increased premium on domain general executive functions such as attention and working memory due to task difficulty.

In the context of speech production related task activity, we note that the sensitization resulted in identifying true positive activity in L-PMd only in the young cohort. The old cohort did not show significant L-PMd activity although they were generating words, but previous work (Wierenga et al., 2008; Meinzer et al., 2012b) has shown that the young and old cohort recruit different language eloquent brain areas to accomplish the task. Across all 3 segments (standard or sensitized BOLD maps) we did not observe significant group differences which is not necessarily a negative finding as other aging studies (Restom et al., 2007) have also reported similar results. Restom et al. quantified task-induced change in CBF and CMRO2 (i.e., %ΔCBF and %ΔCMRO2, respectively) and did not find group differences in BOLD activity because the age-related increase in %ΔCBF was not accompanied by a similar increase in %ΔCMRO2. Another important influence on CBF is baseline hematocrit (Hct), especially since there is a noticeable inter-subject variability (Yang et al., 2014) that accounts for significant amount of variance in visual task fMRI activation (Xu et al., 2018). How these physiologic factors interplay in aging-related task fMRI signals is not yet clear and needs further investigation.

Although the predominant view in aging fMRI literature is that aging results in an increased frontal activation, and posterior to anterior shift in activity, our results contradicts such a view. A recent aging study using multivariate analysis also did not support posterior to anterior shift, and found that age-related increase in frontal activity possessed less information about the cognitive outcome; which they interpreted as decreased specificity and efficiency of neural responses (Morcom and Henson, 2018). Given that our task-specific sensitized activity in older groups did not show an increase in frontal activity, we hypothesize that, taking into account the age-related decrease in frontal CBF, decreases in frontal BOLD activity could be due to reduced specificity and efficiency of de-differentiated neural responses, which needs further investigation.

### Limitations and Future Work

The task and MR protocol design for the current study was different from previous category member generation fMRI studies (Meinzer et al., 2012b; Nocera et al., 2017). In our study, the word stimuli were presented every 4 s, which is relatively shorter than previous studies. Because the stimuli were presented more rapidly than in previous studies, activation of intention-attention components is expected since our task involves attending to externally cued word stimuli followed by intentional initiation of word production. This could explain that in addition to observing domain-specific language areas (see Figure 5) our data shows very prominent domain-general related activity in DMN and fronto-parietal areas involving intention and attention aspects of language processing (Crosson et al., 2007). For the ASL sequence, we implemented a PLD of 1800 ms which may have underestimated the CBF in the older group exhibiting longer arterial transit times. However, few clinical reports describe CBF estimation with longer labeling times, and so we based our ASL sequence optimization on the recommendation from a recent white paper (Alsop et al., 2015). Furthermore, a PLD = 1800 ms represents a compromise between signal-to-noise ratio (SNR) loss from using a PLD much longer than the blood T1 at 3T, and using a PLD that is long enough for the blood to arrive in the tissue. Also, in order to achieve a reasonably small TR of 4 s for ASL sequence, we had to compromise on the whole-brain coverage (i.e., cerebellum was not covered). Future work is necessary to improve the ASL coverage and also increase the PLD safely to ≥2000 ms to better account for aging-related delays in arterial transit time while still maintaining fidelity of the difference signal. Also, given that the difference-based ASL signal is very sensitive to motion, future work should also incorporate background suppression techniques that allows for substantial improvement in the ASL-related SNR (Alsop et al., 2015).

We note that our z-transformation resulted in an approximated normal distribution with slight skewedness (see Supplementary Section S5). The skewedness could be stemming from less stable variance as its estimation was dependent on the number of tent functions and the block length. It should be noted that we had a sufficiently long block of 48 s and we employed 5 tent functions in each segment. Further, a normality test (Kolmogorov–Smirnov) indicated that both standard (KS = 0.163, *p* > 0.15) and sensitized (KS = 0.153, *p* > 0.15) Z (AUC) data from L-PMd were normally distributed. Therefore, we believe that the minimal skewedness in z-transformed AUC should not negatively impact the group analyses results. Further work is necessary to explore whether additional transformation such as Box-Cox transformation (Box and Cox, 1964) can aide in obtaining a more perfect standardized normal distribution.

In this study, we focused on combining task fMRI with ASL to remove the effects of variability of baseline CBF from task-BOLD activity and thereby increasing the BOLD-behavior associations. However, this approach does not dissociate task-induced neural activity from task-induced CBF, and thus future studies should incorporate dual echo based ASL techniques (Wong et al., 1998) that allow acquiring simultaneous task-BOLD and task-CBF measurements. Cerebrovascular reactivity (CVR) is a technique that is sensitive to vascular compliance and previous aging-related studies have shown increased sensitivity to task-specific activity by normalizing the task fMRI signals using CVR (Liu et al., 2013). The relationship between CVR and CBF in aging and neurodegenerative diseases is not well understood. By better delineating the CBF-CVR relationship in age and disease, it will become more apparent if BOLD-correction using only CBF is as effective as CVR-based BOLD correction since CVR data collection in some populations can be challenging. Given that our results show that the achieved power for task fMRI sensitization varies across brain areas, from a pragmatic viewpoint it is perhaps prudent to consider a judicious approach for how to power a study. In other words, given that fMRI studies can be expensive (cost-wise, time, and patient burden), recruiting hundreds of subjects based on power estimation from one specific area might be an overkill of resources as the required subjects might be very few based on the estimated power from other critical brain areas. By assessing the feasibility of this methodology with a small sample size, the observed stability and achieved power is moderate, and has provided a recommendation of sample size (*N* = 40) for important brain areas (such as L-PMd) to successfully implement the proposed methodology in language fMRI studies.

## Conclusion

In summary, we demonstrate that covarying out the baseline CBF from task BOLD fMRI sensitizes the task-BOLD signal to associated behavior in an aging model. Such an approach enhances the task-specificity (removal of false positive activation) as well as the task-sensitivity (detecting false negative activation), enabling more precise conclusions from the data. Our preliminary data suggests that although the predictability of the proposed sensitization approach is better than the other approaches (i.e., standard or normalization by division approach), its stability in such prediction is not significantly different from the other approaches. This suggests that further work with increased sample size and with different task designs need to be implemented for further validation and methodological improvement. From an aging perspective, our data suggests that there is an aging-related delay in blocked-trial hemodynamic response, likely due to compromised vascular compliance. Finally, this study elucidates domain-general aspects of semantic category member generation and the underlying neurophysiological differences due to aging.

## Data Availability Statement

The datasets generated for this study are available on request to the corresponding author.

## Ethics Statement

This study was carried out in accordance with the recommendations of the Emory University IRB and the VA R&D Review committees with written informed consent from all subjects. All subjects gave written informed consent in accordance with the Declaration of Helsinki. The protocol was approved by the Emory University IRB and the VA R&D Review committees.

## Author Contributions

VK, LK, and BC: conceptualization of the study and methodological development. LK, VK, KG, and DQ: MR sequence optimization. BC, ST, and JD: task design. KH, BJ, SR, VK, LK, and JD: MR data collection. KH, JD, SR, AR, KMM, and JN: subject screening. VK, LK, BJ, KM, KG, and SK: data analysis. VK, LK, BC, and JN: manuscript writing and editing.

## Conflict of Interest

The authors declare that the research was conducted in the absence of any commercial or financial relationships that could be construed as a potential conflict of interest.

## Footnotes

1 www.itksnap.org
