# Supplementary material for: Correcting Task fMRI Signals for Variability in Baseline CBF Improves BOLD-Behavior Relationships: A Feasibility Study in an Aging Model
Source: Front Neurosci. 2020 Apr 30;14:336. doi: 10.3389/fnins.2020.00336 (PMC7205008; doi:10.3389/fnins.2020.00336)
Supplement: Supplementary file 1 [file Data_Sheet_1.docx]

**Supplementary Methods**

**S1.** FBIRN criteria for quality assurance of BOLD EPI images

Table below describes the FBIRN quality assurance criteria that were utilized to include/exclude dataset for further preprocessing. A detailed description of FBIRN and the selected criteria can be found elsewhere (Keator DB et al, 2016).

Table S1 : Summary of FBIRN criteria for quality inspection of BOLD EPI data sets. Note: |z| = absolute value of z-transformed signal intensity; FWHM = full-width half maxima; SFNR = signal fluctuation to noise ratio; std = standard deviation.

| **FBIRN criteria** | **Purpose** | **Threshold** |
| --- | --- | --- |
| Volume mean (masked and detrended) | Determine the changes in image signal intensity due to head motion. | Discard if \|z\|> 3 std |
| Running difference ("velocity") volume means (masked and detrended) | Determine the through volume change in image intensity due to spikes induced by gradients and RF field inhomogeneities. | Discard if number of ‘spiky’ volumes > 1% of total number of volumes in each run (which is 78) |
| Center of mass in X/Y/Z direction (masked and detrended) | Determine the head motion translation in X/Y/Z directions | Discard if \|z\|> 3 std |
| FWHM in X/Y/Z dimension | Estimate the kernel size of smoothing applied by the scanner during data acquisition | Discard if the estimated FWHM > 1 std |
| SFNR (detrended) | Estimate the temporal stability of the time series | Discard if the SFNR for a given run is 2 std below the mean across the 3 runs |

**S2.** Age correction of CBF values

Though not implemented in the final model, it was important to determine if age should be removed from the CBF values prior to neuro-sensitization. To remove age from baseline CBF values, the following steps were followed:

*Step 1:* Find the coefficients (D=slope and C=intercept) describing the relationships between CBF from *j^th^* voxel and Age:

$${CBF}_{measured}^{j}=C+D\cdot Age+ \varepsilon^{j}$$

Where ε^j^ is the residual for the jth voxel.

*Step 2:* Extract the age-corrected CBF for *j^th^* voxel by subtracting the predicted value (Step 2) from the measured value, thereby removing the influence of one on the other:

$${CBF}_{age-corrected}^{j}={CBF}_{measured}^{j}-\left( C+D\cdot Age \right)$$

Finally, the age-corrected CBF was tested in the neuro-sensitization scheme as follows:

*Step 3:* Follow the neuro-sensitization steps outlined in the main text, but with age-corrected CBF (from Step 3) as the predictor:

$${Z(AUC)}_{measured,i}^{j}=E_{i}+F_{i}\cdot{CBF}_{age-corrected}^{j}+ e_{i}^{j}$$

Where, ‘*e*’ represents the error term or the residual

$${Z(AUC)}_{un-sensitized,i}^{j}={Z(AUC)}_{measured,i}^{j}-\left( E_{i}+F_{i}\cdot{CBF}_{age-corrected}^{j} \right)$$

**S3**. Mean and age effects on task fMRI and CBF maps from representative areas

The table below demonstrates the average ± standard deviation for BOLD activity (both standard and senstitized) and CBF for the three areas (shown in Table-3) that demonstrated improvements in BOLD-behavior relationship due to sensitization.

Table S2 : Summary of mean and age effects on task fMRI and CBF maps from areas that showed improvements in BOLD-behavior relationship. Note : std = standard deviation ; Y = young ; O = old ; BOLD_std_ = standard BOLD analysis ; and BOLD_sens_ = BOLD sensitized for CBF variability

|  |  | BOLD_std_  mean ± std | | BOLD_sens_  mean ± std | | CBF  mean ± std | |
| --- | --- | --- | --- | --- | --- | --- | --- |
| Area | Segment | Y | O | Y | O | Y | O |
| L-PMd | 2 | 1.06 ± 1.19 | 0.79 ± 1.52 | 1.23 ± 1.06 | 0.6 ± 1.41 | 51.42 ± 11.46 | 39.83 ± 12.97 |
| R-postCent | 2 | 1.55 ± 1.31 | 1.27 ± 1.09 | 1.6 ± 1.22 | 1.23 ± 1.12 | 47.92 ± 10.85 | 39.63 ± 12.69 |
| PCUN/PCC | 2 | 0.66 ± 2.2 | 1.15 ± 1.08 | 0.97 ± 1.96 | 0.89 ± 1.06 | 50.87 ± 9.58 | 39.99 ± 10.65 |

**S4**. Comparison of group maps between proposed group level co-variate normalization approach with the standard BOLD analysis and normalization by division approach.

Figure S1 : A mosaic view comparing within-group significant (voxel-wise p<0.01, FWE corrected) maps for standard, normalization by division (i.e. Div. By CBF) and the proposed sensitization approach for the young group. The Segment 1 BOLD activity is shown here. The scaling of z-scores and color map is similar to that described in the main text. The solid red circles show false positives retained in both standard and normalization by division approach. The dashed yellow circles denote task-specific areas that were retained in our proposed approach but removed in either of the other approaches.

Figure S2 : A mosaic view comparing within-group significant (voxel-wise p<0.01, FWE corrected) maps for standard, normalization by division (i.e. Div. By CBF) and the proposed sensitization approach for the Old group. The BOLD activity is quantified separately for each segment (which in this case is segment-1). The scaling of z-scores and color map is similar to that described in the main text. The solid circles show false positives retained in both standard and normalization by division approach.

Figure S3 : A mosaic view comparing within-group significant (voxel-wise p<0.01, FWE corrected) maps for standard, normalization by division (i.e. Div. By CBF) and the proposed sensitization approach for the young group. The BOLD activity is quantified separately for each segment (which in this case is segment-2). The scaling of z-scores and color map is similar to that described in the main text. The dashed yellow circles denote task-specific areas that were retained in our proposed approach but removed in either of the other approaches.

Figure S4 : A mosaic view comparing within-group significant (voxel-wise p<0.01, FWE corrected) maps for standard, normalization by division (i.e. Div. By CBF) and the proposed sensitization approach for the old group. The BOLD activity is quantified separately for each segment (which in this case is segment-2). The scaling of z-scores and color map is similar to that described in the main text. The solid circles show false positives retained in both standard and sensitized but not in the normalization by division approach. The dashed yellow circles denote task-specific areas that were retained in our proposed approach but removed in either of the other approaches.

Figure S5 : A mosaic view comparing within-group significant (voxel-wise p<0.01, FWE corrected) maps for standard, normalization by division (i.e. Div. By CBF) and the proposed sensitization approach for the young group. The BOLD activity is quantified separately for each segment (which in this case is segment-3). The scaling of z-scores and color map is similar to that described in the main text.

Figure S6 : A mosaic view comparing within-group significant (voxel-wise p<0.01, FWE corrected) maps for standard, normalization by division (i.e. Div. By CBF) and the proposed sensitization approach for the old group. The BOLD activity is quantified separately for each segment (which in this case is segment-3). The scaling of z-scores and color map is similar to that described in the main text. The dashed yellow circles denote task-specific areas that were retained in our proposed approach but removed in either of the other approaches.

**S5**. Z-transformation of Area under the Curve (AUC) with a representative data

As described in methods section (Task-fMRI Pre-processing), the area under the curve (AUC) for each voxel from a given segment of the hemodynamic response function (HRF) was z-transformed using the formula: $\frac{\left( {AUC}_{i}-\mu_{i} \right)}{\sigma_{i}}$ where μ_i_ and σ_i_ are the mean and standard deviation of the task-beta coefficients for the *i*^th^ voxel of an individual subject. In order to confirm that the data was indeed z-transformed, Figure below show the histogram and fitted normal distribution (solid red line) for AUC and Z(AUC) data from a representative subject’s HRF segment. We note that our z-transformation resulted in an approximated normal distribution with slight skewness. The variance estimation was dependent on the number of tent functions (5 per segment) and the block length (48sec), and may partly be responsible for the observed skew. Nevertheless, we believe that the slight skew in z-transformed AUC does not negatively impact the group analyses results.

Figure S7 : Histogram of voxel-wise AUC and Z(AUC) data (in blue) along with fitted normal distribution (solid red line) from a representative subject’s HRF segment. Note that after z-transformation, the μ=0.2 and σ=1.5.

**S6**. Predictability of behavior across the methodologies

Figure S8: Box plots for predictability of behavior across the three different methodologies (standard shown in red, sensitized shown in black and normalization by division (i.e., DivByCBF) in magenta) quantified from 3 different brain areas. Note that asterisk denotes p<=0.0001 and diamond symbol denotes p=0.0001 significant difference.

**S7**. Stability in prediction of behavior across the methodologies

Figure S9: Box plots depicting the cros-validation (CV) error in predicting behavior across the three different methodologies (standard shown in red, sensitized shown in black and normalization by division (i.e., DivByCBF) in magenta). No significant differences were observed between the methodologies. Note: the grey dots denotes data point from an individual subject,

**S8**. Predicting behavior using multiple linear regression approach

With the premise that BOLD is a neurovascular signal, a very simple approach to model the neuro-vascular contributions to BOLD is:

${BOLD}_{standard}=N+V+\varepsilon$	-------------- (i)

where, N= task-induced neural activity, V= baseline vascular contribution, and ε = non-physiological noise contribution, all from a unit voxel.

Then,

${BOLD}_{sensitized}=N={BOLD}_{standard}-\left( V \right)$	------------ (ii)

In the proposed sensitization model (i.e., Equation-1 in the manuscript), we have:

${BOLD}_{standard}=A+B*CBF+res$	-------------- (iii)

where, A and B are coefficients and res is the residual. Thus,

$res={BOLD}_{standard}-\left( A+B*CBF \right)$	------------ (iv)

Comparing equations (ii) and (iv), notice that $V= A+B*CBF$

or $V=\left( A+B*CBF \right)$which is meaningful as CBF predominantly encodes baseline vascular contribution. Also, comparing equations (ii) and (iv) note that the residual in equation (iv) is equivalent to sensitized BOLD (i.e. task-induced neural activity).

With the above background, now if we were to model the behavior using multiple linear regression incorporating both standard and sensitized BOLD, we will have:

$Behavior=D+\left( E*{BOLD}_{standard} \right)+\left( F*{BOLD}_{sensitized} \right)+\eta$ ---------- (v)

If we plug-in equations (i) and (ii) into equation (v), then we have:

$Behavior=D+ E*\left( N+V+\varepsilon\right)+F*N+\eta$	--------- (vi)

$Behavior=D+\left( E+F \right)*N+E*\left( V+\varepsilon\right)+\eta$	-------- (vii)

In equation (vii), for simplicity if we substitute E=F=1 then,

$Behavior=D+2N+V+ \left( \varepsilon+\eta\right)$	---------- (viii)

In the simplified equation (viii), note that the behavior is mischaracterized with 2 * task-induced neural activity which is not physiologically meaningful and our results section (Sensitization and BOLD:Behavior Relationships) indicate the baseline vascular component (i.e., V) does not relate with behavior. Therefore, we believe that the multiple linear regression model (for simultaneously incorporating both ZAUC_standard_ and ZAUC_sensitized_ to predict Behavior) will not add clarity in accurately characterizing the sensitization of task-BOLD signal.
